# Determining and quantifying the historical traces of spatial land arrangements in rural landscapes of Central and Eastern Europe

**DOI:** 10.1038/s41598-021-02892-x

**Published:** 2021-12-03

**Authors:** Barbara Prus, Małgorzata Dudzińska, Stanisław Bacior

**Affiliations:** 1 Department of Land Management and Landscape Architecture, Faculty of Environmental Engineering and Land Surveying, University Agriculture, Balicka str. 253c, 30-149 Kraków, Poland; 2 Institute of Geography and Land Management, Faculty of Geoengineering, University of Warmia and Mazury, Prawocheńskiego str. 15, 10-719 Olsztyn, Poland; 3 Department of Agricultural Land Surveying, Cadastre and Photogrammetry, Faculty of Environmental Engineering and Land Surveying, University of Agriculture, Balicka str. 253a, 30-149 Kraków, Poland

**Keywords:** Civil engineering, Environmental impact

## Abstract

The article attempts to define and determine the intangible components of cultural heritage related to the spatial structure of land in a comprehensive way using computational methods. The components were quantified and a method of empirical evaluation of landscape durability was proposed for agricultural areas of significant cultural and historical value with an evident mosaic structure of fields, baulks, ponds, meadows, and forests. This method allows us to identify places more resistant to political transformation and those with greater cultural potential. The paper proposed an integrated approach to the measuring of the degree of preservation of spatial arrangements in the landscape based on a set of objects that describe the spatial land structure. The article classifies areas by the degree of preservation of rural spatial arrangements of land. The spatial analysis employed facilitated a synthetic quantification of the multi-criteria process. Three groups of factors were used: spatial assessment of land-cover type persistence (u), agricultural land structure persistence (w), and persistence of settlement buildings (z). The final results pinpointed areas in need of strategic intervention to sufficiently protect the rural cultural heritage, properly consider them in zoning planning, and ensure their sustainable development. The proposed tool can be used to monitor the degree of changes in the landscape layout structure when multiple time points are analysed as well.

## Introduction

Nearly 50% of Europe's area is covered in agricultural land that builds characteristic agrarian landscapes. From the historical point of view, Europe has visually open and large-scale open-field landscapes and small-scale, enclosed ‘bocage’ landscapes^1^. The Institute for European Environmental Policy defines traditional agricultural landscapes as those that emerged from historical, meaning native, methods of land management, where traditional cultivation defined the dominant features of the cultural landscape. The characteristic feature of these agricultural systems is the distribution and mosaic arrangement of agricultural parcels characteristic of each region that determine how the landscape is perceived through its aesthetic qualities and even agrobiodiversity^2^.

Many European countries were transformed in the twentieth century. In Central and Eastern Europe, the transformations were rapid and intensive, such as changes in the landscape structure due to the collectivisation of land in the former Soviet Union and Eastern Block^1,2^.

Research on historical agricultural landscapes indicates periods of relative stability entwined with times of transformation^1^.

European rural landscapes are being transformed due to climate change or changes in agriculture and public policy combined with urbanisation^3^. The changes affect the agricultural space^4^ and sociocultural identity of rural areas^5^. Every landscape has its unique history and individual features. In addition, landscape structures and field layouts evolve pressed by diverse factors^6^. Bürgi et al. grouped drivers of agricultural landscape change into five main forces^7^:

- socio-economic
- political, embodied in political agendas, laws, and politics
- technological, such as the effects of infrastructure expansion (including agricultural management and consolidation projects or the expansion of the railway transport in the nineteenth century)
- natural, consisting of settlement factors (soil parameters) and natural disturbances (from slides to climate change)
- cultural drivers as the most complex dimension of landscape-shaping factors.

Before the Second World War, Eastern and East-Central Europe faced significant political and economic changes^8^. Poland until 1989 was under the influence of nationalisation and centrally-planned economy. During this period environmental and landscape changes weren’t so significant, which is due to, among other things, economic stagnation. After that date Poland transited from socialist planning to a market-oriented economy following the collapse of the USSR, which resulted in several structural changes^9,10^.

Although in the past, development focused on new geographical areas, today, previously developed areas are used increasingly often.

Cultural heritage, the patrimony of multiple generations is an important component of transformed space on which the progress exerts pressure^11^. Cultural as well as social and ecological heritage is traditionally regarded as a non-economic factor of spatial development^12^. However, treated as a resource and properly managed, it can be transformed into capital that can play an important role in sustainable development strategies, both at the local and national level. Cultural heritage as a particular type of resource of local communities has become an appreciated market asset^13–16^. It is an important part of the identity and driver of social integration^17,18^.

Agricultural landscapes can be considered cultural heritage. Even though their historic values differ, each can be assigned value and appeal, if only on a local scale. They can provide a foundation for cultural, educational, economic, or tourist activities and be a backbone for the local identity^19^. Researchers identify heritage resources even today with the progress of the work varying by site. While global cultural heritage assets have been identified to a large extent, regional ones in ‘small homelands’ call for further effort, particularly in agricultural areas. They should be identified immediately because the progress of civilisation might contribute to their irreversible loss. Excessive transformation can render an area not eligible for the cultural heritage list.

The awareness of the need for rational management of cultural heritage assets and their preservation for future generations has been discussed extensively^20^. Cultural heritage may not be directly perceivable in the physical landscape^21^. It is, therefore, impossible to capture its transformations without a spatial analysis. In many regions in Europe the landscape includes spatial arrangements of villages, the fields, meadows, rivers, lakes, hills, and forests around it as well as products of ages-long human impact on the environment. All these components can be arranged in various ways depending on the region in the world and topography. They should be protected, mainly by preserving their characteristics. With these resources identified, local governments gain new tools to perform their statutory obligations and implement solutions to stimulate the development in these areas, for example^22^. Identification of places where cultural heritage assets are located is vital for sustainable rural development. It stimulates more comprehensive growth of the areas. Hence the need to search for more effective ways of identifying cultural heritage assets and their quantitative analysis.

The knowledge about places where the characteristic historical spatial structure of land has been preserved is one of the inputs necessary to prevent the loss of culturally valuable agricultural landscapes.

The aim of the study is to determine the intangible i.e. to propose a new methodology for identifying and measuring the degree of persistence of historically formed spatial arrangements of rural areas not only in the agricultural landscapes of Poland but also in mosaic agricultural land found in Central and Eastern Europe. These are often long and narrow parcels where crops on arable land alternate with grassland, permanent crops, and stands of trees among fields, called complex cultivation patterns. Such management and agricultural use of land were actively influenced by human activity, parcel divisions, and legal mechanisms allowing inheritance by multiple people^10^. The proposed method is an integrated, quick, and precise quantitative analysis-based approach to assessing the degree of preservation of spatial arrangements in the landscape based on a measurable set of elements that describe the spatial land structure. The method can identify enclaves of the traditional landscape with unique traditional spatial arrangements of agricultural landscape structures that change the slowest or the least. Solymosi^2^ defined such places as cultural landscape hotspots. Identifying such sites is the starting point for further research, qualitative and quantitative both, on the preservation of cultural heritage.

The proposed typology of degrees of preservation and persistence of traditional spatial arrangements was based on a four-point scale. The taxonomic methods employed facilitated a synthetic quantification of the multi-criteria process. Three groups of factors were used: spatial assessment of land-cover type persistence (***u***), agricultural land structure persistence (***w***), and persistence of settlement buildings (***z***). The final results pinpointed areas in need of strategic intervention to sufficiently protect the rural cultural heritage, properly consider them in spatial planning policy, and ensure their sustainable development and protection.

## Theoretical background

### Agricultural landscape

The agricultural landscape has its characteristic features. Boundaries between parcels, baulks, stones, boundary mounds and trees, access roads, characteristic crops, and buffer strips make them unique. Agricultural operations are the primary actions that affect the dynamics of the agricultural landscape^23^. One can notice seasonal changes in the appearance of the landscape. Ploughing, sowing, harvest, hay cutting or silage collection; planting, thinning, or felling forest trees cause changes in colour and composition, dynamics in the landscape, often in a way characteristic of the place^24^.

Changes in the landscape are often perceived as a threat, negative evolution because changes today most often involve loss of diversity, coherence, and identity of existing landscapes^25^. New elements and structures are introduced that look the same regardless of the location.

Agricultural areas are particularly prone to changes caused by variations in demographics, farming (including new crops) intensified land use through innovative agricultural techniques and reforms^26^, or land abandonment resulting in forest succession, on the other hand.

Changes in the agrarian structure, such as consolidation of land, removal of baulks or stands of trees among fields, and disappearance of environmentally valuable land simplify the landscape and render it monotonous. Significant deforestation changes hydrographic conditions, endangering traditional extensive meadows and pastures, peat bogs, and ponds among fields.

Researchers often focus on the landscape and appreciate the need to classify and assess it^27^. They propose the notion of landscape value and classify it^28^. The assessment of landscape, its structural complexity, relations and processes within it are best summarised in the notion of ‘landscape structure’. Kondracki and Richling^29^ defined landscape structure as a ‘complex of components making up the landscape and their interrelations’. A dynamic landscape structure analysis involves the determination of relationships and dependencies among units (the horizontal dimension) and components (vertical dimension). Landscapes usually change through changes in their structural components^30^. Not many researchers looked into the opposite phenomenon, the persistence of the landscape^7^. Landscape persistence is the contradiction of landscape change. Landscape persistence is bound up with identity and landscape values^25,31^. It is often related to the notion of ‘traditional landscape’^32–36^. Persistence is also one of the attributes of cultural characteristic and valuation of the landscape^21^ as well as an important criterion for identifying traditional cultural landscapes^37–40^. In general, the literature describes traditional landscapes as those where farming consumes low external resources is dominated by relatively small (family) holdings. One of the main threats to the cultural value of these landscapes is related directly to the intensification of land use^41^.

The historical value of the traditional agricultural landscape is assessed based on its characteristic features: (a) the small-scale structure of the plot division is preserved; (b) the presence of original forms of anthropogenic relief (baulks); (c) unaltered land use; (d) employment of some traditional farming technologies^38^.

Tieskens et al. presented characterisation of European cultural landscapes based on the prevalence of three key dimensions of cultural landscapes: landscape structure, management intensity, value and meaning. They integrated and mapped the three dimensions into a continuous ‘cultural landscape index’ that facilitates a qualitative description of Europe's rural landscapes^21^. Jepsen et al. measured forest structure and its cultural value as related to its persistence understood as length of the period that the forest has been covering the area^42^. Agricultural landscape attributes that describe mosaic land cover, historical buildings, or the presence of livestock generally receive the highest value assessment^43^.

### Characteristics of spatial arrangements in Polish agricultural areas

Eastern and south-eastern Poland, with its relatively extensive agriculture, offers particularly valuable agricultural landscapes exhibiting high biodiversity and rich genetic pools^44^. The landscapes of central, eastern, and southern Poland have small traditional holdings with mosaics of fields, baulks, ponds, meadows, and forests. The natural environment of most rural areas demonstrates an evident mosaic structure shaping the traditional landscape^44^.

The next landscape category is the heterogeneous, disharmonious landscape. These have large holdings practising intensive production (large fields without baulks, trees, or natural infrastructure) mixed with small, traditional holdings, small processing plants, or recreation and tourism sites.

Poland also has areas where the balance has been disturbed by human pressure, simplifying landscape structure and reducing biodiversity. A good example is numerous sites in north-western Poland where rural areas are considered mainly production zones with concentrated, large-area, intensive holdings, large areas of greenhouse or growing tunnel production, or intensive pig farming, while the economic growth is fuelled by the degradation of natural heritage^45^.

A mosaic farm structure typical of certain regions results in a striped or patchwork layouts of plots.

## Materials and methods

### Research organisation

To devise the methodology, the authors conducted empirical qualitative research to find a list of indices for spatial arrangement and quantitative research in order to measure them. This effort resulted in a classification of places with different degrees of spatial arrangement transformation.

To identify factors for assessing rural landscape layouts characteristic of land spatial arrangements of Central and Eastern Europe, the authors reviewed the literature, focusing on research techniques employed to evaluate landscape layouts, materials for analysis^46–48^, determination of indicators for assessing changes and persistence of the landscape and its structure^40^, and changes in land use. They also looked into the mosaic aspect of the agricultural landscape^24^.

The most common methods for assessing the variation in the spatial landscape structure are methods based on historical maps and photography analysis. The landscape structure is then estimated quantitatively with a set of landscape indicators^49^. The selection of characteristic variables for identifying landscape layouts is adjusted to the available area-covering spatial data at the given resolution^50^ and the objective of the analysis. According to Simensen^50^, ‘Landscape metrics are focused on the description of the geometric and spatial properties of categorical map patterns and allow the characterisation and the investigation of land-use change processes’. The following indices are used most often: the total area, number of patches, perimeter-area ratio^51–53^, mean core area, Euclidean nearest-neighbour distance, the mean shape index, total edge^46^, landscape heterogeneity index, patch shape index, neighbour patch context, and the nearest neighbour distance index^53^. Other indices in the literature represent the general level of land-use change, like the binary change index^54^. Some researchers linked landscape structure to land ownership^21,55,56^. The shape of the field often results not only from the boundary and shape of the area of uniform land-use type but also from plot or parcel boundaries. Baulks, fencing, or lines of trees enrich the landscape and contribute to its increased mosaic. Note that the plot structure has the most significant impact on the seasonal appearance of the agricultural landscape. Baessler and Klotz^49^ and Stanfield^56^ looked into the aspect of ownership structure. Benoît^23^ pointed out the relationship between agricultural practices and landscape dynamics. The spatial structure of parcels is assessed based on parcel area, shape, and elongation indices, holding's parcel configuration^57^, or the patchwork (defined as a layout of plots or parcels separated by baulks etc.).

The research actions were planned and conducted to develop a methodology for identifying groups of areas exhibiting different degrees of preservation of spatial arrangements. This approach is vital for the preservation of cultural heritage assets that are under the pressure of such factors as urbanisation, globalisation, increasingly notorious natural disasters, or impact of the special pandemic circumstances today. The recognition methodology can be used as a tool for identifying such places to implement repair schemes or future local policies. A preserved historical layout of a locality is a testimony to how its long-gone residents shaped the place. Being historical development layouts, arrangements of buildings, complexes of houses, or hamlets are often listed as historical heritage in and of themselves. Research seldom focuses on the surroundings of these sites. No comprehensive research has been done on heritage assets to offer an integrated index representing the degree of preservation of the land structure, land-use types, and buildings.

Historical spatial arrangements, particularly rural ones, are often inconspicuous, and the local community (authorities) is unaware of their existence and location and fails to exploit them for local development. Cultural heritage is analysed on various levels of detail, but only a local analysis can capture historical spatial arrangement.

The proposed method can be applied to rural areas in Central and Eastern Europe but also in selected agricultural landscapes areas in the world where the spatial arrangement of land is similar. Thus it is not limited by the size, location, population, or economic status of the site. The methodology involves quasi-automatic data processing. One of its requirements is the availability of historical and modern maps for comparative analysis. It focuses on the use of geoinformatics to produce thematic maps with identified areas that are uniform in terms of the degree of preservation and persistence of historical layouts. Neither academic literature nor practical guides propose this approach to space assessment, especially regarding Poland.

The availability of data that describe the historical value of agricultural landscapes with the indicators from groups: spatial assessment of land cover type persistence, agricultural land structure persistence, and persistence of building location in space presented above is a technical limitation of the proposed method. The limitation is particularly apparent for maps that should present both land use structure and ownership structure. Moreover, the arrangement of the mosaics (historical and present state) should have similar characteristics as shown with the case study, that is, parcels shapes such as rectangles, rhombi, or other elongated figures. Such conditions can be identified mainly in areas where individuals own the land instead of state-controlled farms or associations, which cause changes in the agrarian structure by definition^10^.

### A method for classifying the persistence of layouts

The analysis of cultural landscape change in Europe demonstrated that the preservation of traditional structures of the cultural landscape is affected by three main factors. These are isolation, difficult geographical situation, and nature of the local community. In-depth research demonstrated that the changes progressed faster in more developed areas closer to local centres, along roads. The trigger for the changes was a society with access to modern communications and infrastructure and additional funding, resulting in the ‘industrialisation’ of agriculture. Nevertheless, traditional agricultural structures and systems persisted in places where farming was a source of income or satisfied household consumption with surplus produce being sold, i.e. most often in the poorest families and ageing parts of the population^2^.

During the study it was assumed that rural landscapes in Central and Eastern Europe, the function of which did not change in a specific time, are more likely to contain cultural heritage assets, as researched i.e. Solymosi et al.^2^. She expected land-use transformation and changes in the possession structure to contribute to the loss of at least part of the cultural heritage potential. While aware of the fact that land use could change several times and return to the initial type over the analysed period, the authors considered such a possibility a marginal risk because the area has been rural^58^.

**Figure 1:**
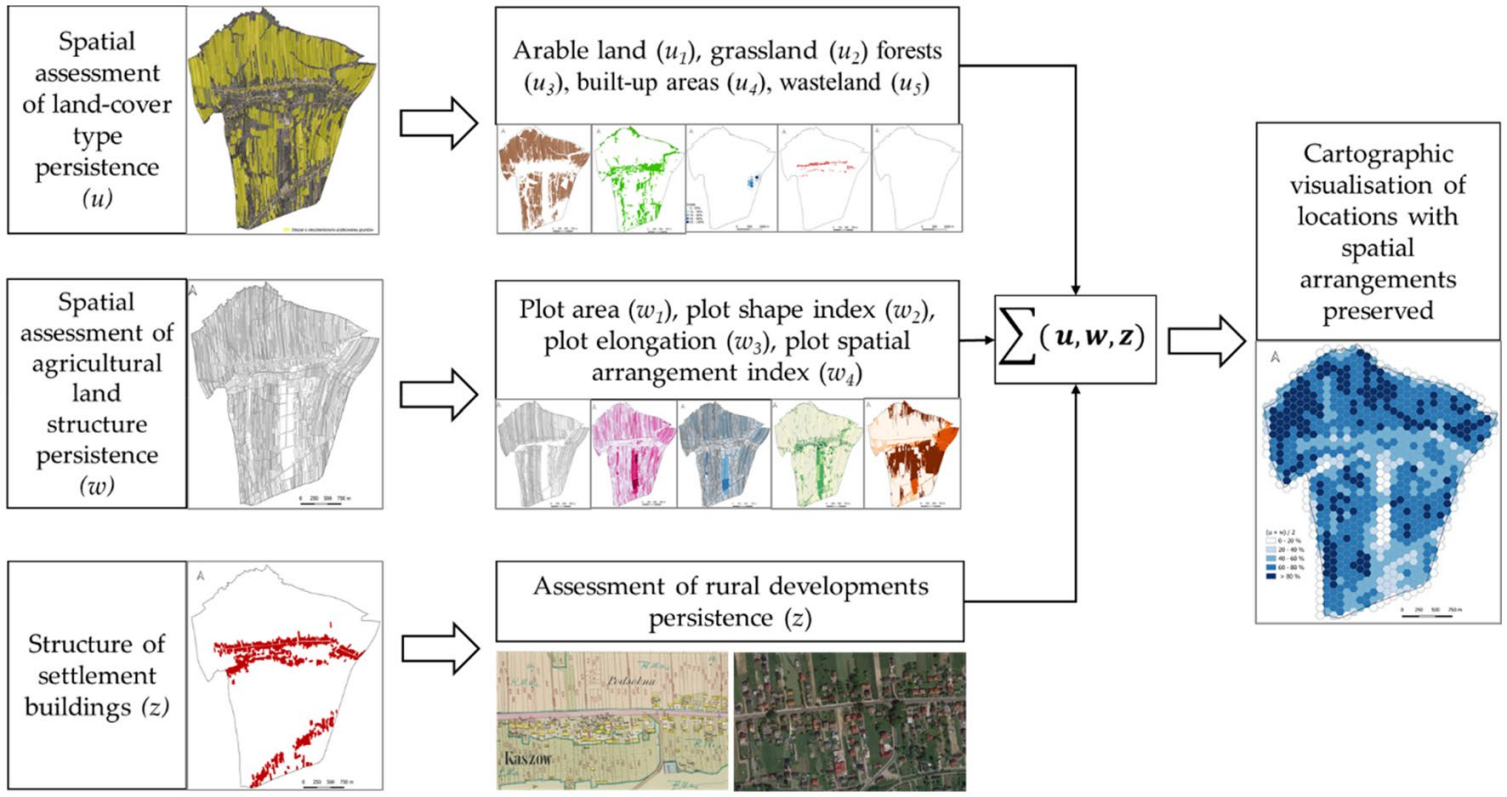
Research stages scheme.

The assessment involved three primary domains (see Fig. 1): (1) spatial assessment of land-cover type persistence; (2) spatial assessment of agricultural land structure persistence; and (3) structure of settlement buildings.

#### Spatial assessment of land-cover type persistence-Land-use structure index

Landscape persistence depends on the types of land cover that remain unaltered for some time^40^. Land-cover change (by land-cover types) was assessed using the land-use structure persistence equation ‘***u***’ (1).

1 $${\varvec{u}} = \mathop \sum \limits_{A = 1}^{n} \left( {A_{t0} \cap A_{t1} } \right)$$

where ***u*** the land-use structure persistence index; ***A***_***t0***_ a selected land-use type: arable land, wasteland, grassland, forests, built-up areas in a start time ***t***_**0**_; ***A***_***t1***_ a selected land-use type in end time ***t***_**1**_; ***n*** the number of land-use types per spatial unit. This equation can group areas by the persistence of unchanged land use.

The analysed area was divided into spatial units-hexagons of 1.1652 ha in this case-where the degree of land-use persistence in start time ***t***_**0**_ and end time ***t***_**1**_ was assessed. The arrangement and size of spatial units should be chosen to suit characteristics of the area, its size, development, and spatial variation.

#### Spatial assessment of agricultural land structure persistence

**Figure 2:**
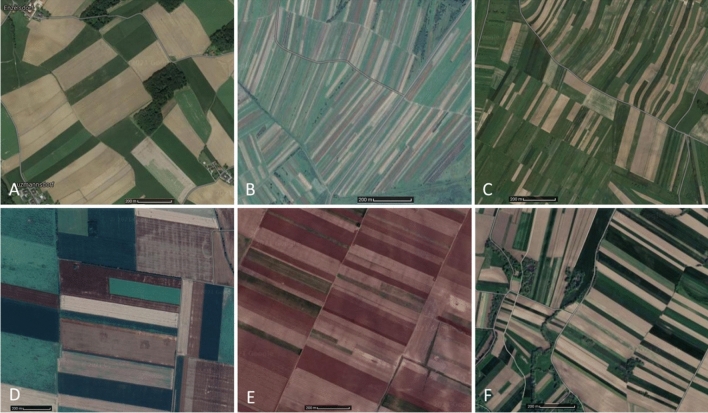
A local aspect of the spatial arrangement of fields (visual interpretation of ***w*** index) at the examples of East and Central Europe (**A**-Austria/Lutzmandorf; **B**-Ukraine/Hruszów; **C**-Poland/Sułoszowa; **D**-Hungary/Csanadapaca; **E**-Romania/Ramnicelu; **F**-Slovenia/Gradisce). Source: google maps.

It is not only the type of land use that shapes the spatial arrangement of a landscape structure. The shape of the field and plot also affects its appearance, particularly the dynamics of the agricultural landscape^23^. Baulks are the most clear boundaries in the landscape (see Fig. 2).

The image created by baulks is called a patchwork of fields. The number and layout of baulks determine the shapes and sizes of patches of land that make up the patchwork (mosaic structure). The mosaic can consist of small, irregular figures, large and regular ones, or long, narrow bands. The baulk is a natural or artificial separation of land patches resulting from multi-generational ownership arrangements. Therefore, the authors considered it necessary to assess the persistence of land layouts in the area. For the research objective, it is the layout of parcels in space, not their size that is important. Hence the importance of comparison of land structure distribution. The statistical procedure was based on total ordering and classification of spatial units (such as hexagons). The possession conditions at start time ***t***_**0**_ covered land parcels that have been transformed into plots at end time ***t***_**1**_.

The proposed method includes four stages.

- *Stage I* is a determination of component indices for land structure assessment. The variables were selected also in four steps. Based on in-depth analysis of literature on land structure assessment^57,59,60^, the authors first selected a set of features that best described the investigated phenomenon. The selection was determined by the measurability, availability, and completeness. Land structure was assessed using plot area ***w***_**1**_, plot shape index ***w***_**2**_, field layout assessment index was derived from the component indices, index of plot elongation ***w***_**3**_, and mean plot axis angle ***w***_**4**_.
- *Stage II*. In the second stage the component indices (***w***_**1**_ to ***w***_**4**_) for a start time ***t***_**0**_ and end time ***t***_**1**_ were calculated. This way, spatial units could be total ordered into categories with similar values of the indices. In this case, the spatial units were hexagons.
  The ***w***_**1**_ index was calculated in hectares directly from the polygon coordinates. Shape index ***w***_**2**_ was calculated using Eq. (2) originally developed for representing landscape fragmentation^61,62:^
  2 $$w2 = \frac{pi}{{2\sqrt {\pi w1} }}$$
  where ***p***_***i***_-the plot perimeter, ***w***_**1**_-the plot area.
  The ***w***_**3**_ index-the plot elongation was calculated using formula (3):
  3 $$w3 = \frac{a}{b}$$
  where ***a***-the shorter side of the plot; ***b***-the longer side of the plot. The larger ***w***_**3**_, the more elongated the parcel shape.
  The ***w***_**4**_ index-the main axis angle is assumed to be the main angle of the polygon. The longest side of the polygon is considered its main axis. It indicates the direction in which plots are positioned in space. This operation used the Calculate Polygon Main Angle tool in ArcGIS.
- *Stage III*. At the third stage, the authors compared the results for start time ***t***_**0**_ and the end time ***t***_**1**_ using intersection (4):
  4 $$W = \mathop \sum \limits_{w = 1}^{m} \left( {W_{t0} \cap W_{t1} } \right)$$
  where ***W*** land structure persistence index; ***W***_***t0***_ component indices for land structure assessment (i.e. area, shape, elongation, and angle) in start time ***t***_**0**_; ***W***_***t1***_ component indices for land structure assessment in end time ***t***_**1**_; ***m*** the number of component indices used. The analysis was performed using a five-point scale (20% steps).
- *Stage IV*. The goal of this step was to construct an integrated index for spatial assessment of the persistence of agricultural land structure. Homogeneous objects were classified with Eq. (5):
  5 $$w = \frac{1}{n}\mathop \sum \limits_{i = 1}^{m} Wi$$
  where ***w*** the integrated index for spatial assessment of persistence of agricultural land structure; ***W***_***i***_ component indices; ***m*** the number of component indices.

#### Spatial assessment of settlement buildings

The assessment of rural development involved the determination of an index of persistence of building distribution (Eq. 6) for two points in start time ***t***_**0**_ and end time ***t***_**1**_.

6 $$Z = \mathop \sum \limits_{B = 1}^{l} \left( {B_{t0} \cap B_{t1} } \right)$$

where ***B***_***t0***_ buildings in start time ***t***_**0**_; ***B***_***t***1_ buildings in time ***t***_1_; ***Z*** building location persistence index; *l* the number of buildings in the area.

This equation can identify buildings that are located in similar places (partially consistent locations) in time ***t***_**0**_ and ***t***_**1**_.

### Materials

Maps are widely used in quantitative analyses of land cover changes in Europe and analysis of the spatial structure of land^63^. They help reach into the history, providing sufficient precision for small- and medium-scale research^64,65^. Austrian cadastral maps are generally considered reliable, detailed, and useful for assessing landscape structure in the former territory of the Austro-Hungarian Empire^66^.

The main sources for reconstructing historical spatial composition of a rural area according to its history were maps created when surveys for the second Galician cadastre called the Franciscan cadastre were conducted in the former Austro-Hungarian Empire (which covered the entire or partial area of twelve Central-European countries, including Austria, Bosnia and Herzegovina, Czechia, Croatia, Hungary, Italy, Poland, Serbia, Slovakia, Slovenia, and Ukraine) and an orthophoto with a Database of Topographic Objects, DTO10k overlay for 2016. The DTO10k includes units of administrative division, transport network, buildings (their location and functions), and complexes of land-use types. The research was carried out in QGIS and ARCGIS using geoprocessing tools as well as data management tools (a combination of attributes according to the location).

The Franciscan Cadastre maps were drawn using a rectangular arrangement of 1 Austrian mile square sheets, so-called triangulation map sheets^67,68^. A smaller scale allowed more details to be recorded. Information provided by the map includes the type of development, layout of development, plot boundaries and numbers, water bodies, vegetation (including its arrangement and forms), spatial arrangements (spatial layouts, links, spatial relations, sequences), composition (such as sightlines, landmarks, emphasis, vantage points and view obstructions, and sets of vantage points). Such information helps analyse the type of development layout and its basic modules, parcellation, plot size with their typical shape and dimensions, width and positioning of buildings relative to streets, layout and density of buildings on a plot and arrangement of fields. They record past boundaries and subdivisions^68^. The map content is certainly abundant and precise. Patterns and colours make up about 150 legend items^69^.

The analysis required a prior development of a key for interpreting map labels and photointerpretation keys. Taking into account the objective of the study, the quality of the Austrian cadastral maps (scale 1:2880), the accuracy of terrain representation on orthophotos, and land-use structure form the DTO10k, the authors had to modify the photointerpretation keys. The modification concerned mostly grassland (recently not divided into meadows and pastures) and built-up areas. Seven land-cover categories for rural areas were identified: arable land, grassland, wasteland, built-up areas, water bodies, roads, and forests.

### Study area

**Figure 3:**
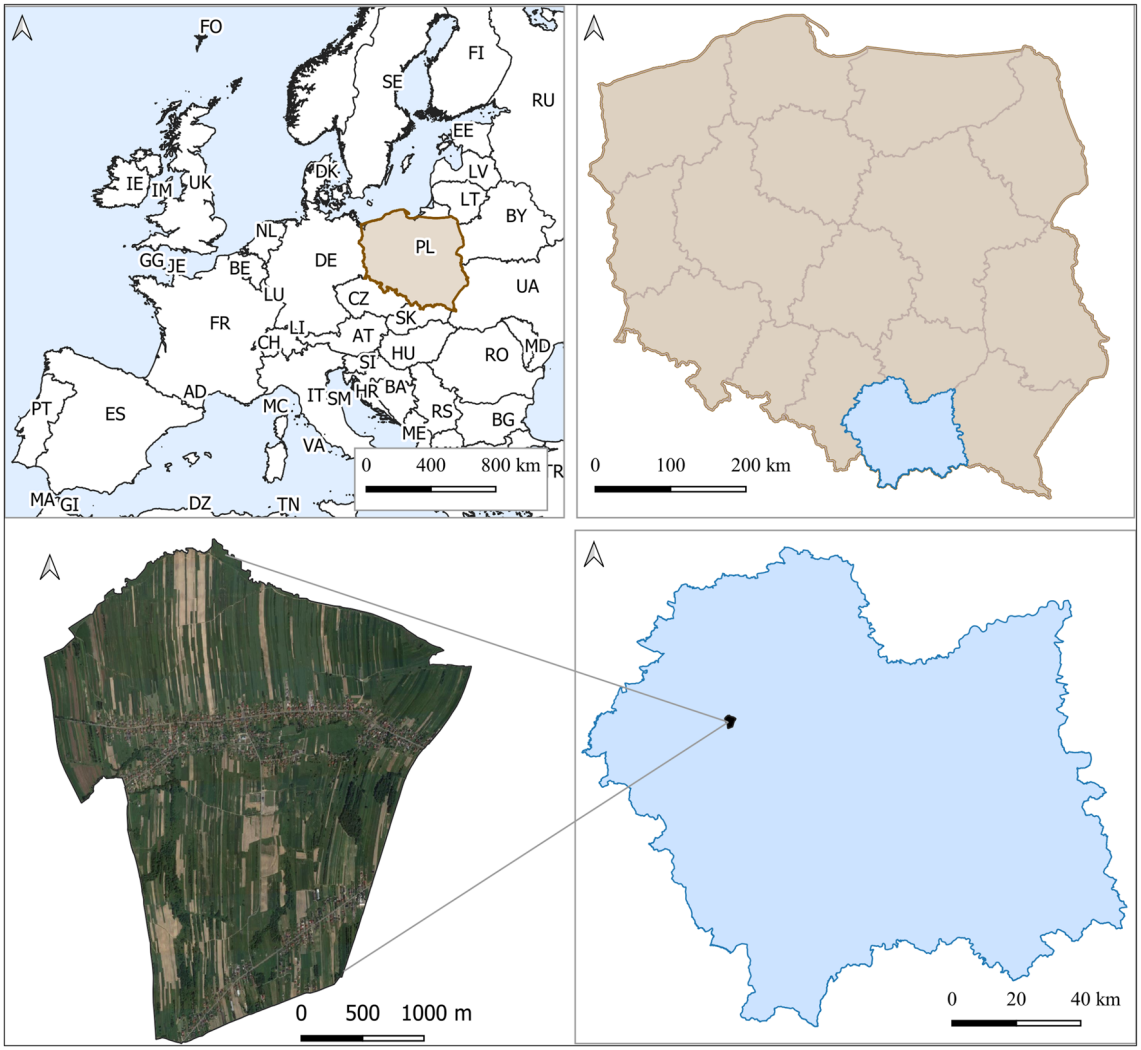
Location of the study area, Kaszów coordinates 552106, 241411-EPSG 2180.

The authors are perfectly familiar with the typical agricultural village of Kaszów, located in the south part of Poland, Europe in Małopolska (Fig. 3), subjected to the investigation selected to measure the degree of preservation of historical rural layouts. This way, it was easier to verify the results and assess the soundness of the approach.

Kaszów is a village established around the twelfth century. To this day, the village has been characterised by a linear housing pattern. In 1869, there were 250 peasant cottages in Kaszów, inhabited by 1,401 people. Over the following years, the population grew steadily to reach 1,523 inhabitants as early as 1880. The place occupied an area of 1,446 Austrian morgens (1 Austrian morgen = 0.56 ha). The land use structure of that time was dominated by arable land (922 morgens), pastures (277 morgens), meadows and gardens (227 morgens), and only a little forest of 20 morgens.

The settlement was located on the transit route, the high road from Kraków to Silesia. It remains a linear village to this day^27^.

## Results

### Spatial assessment of land-cover type persistence (u)

**Figure 4:**
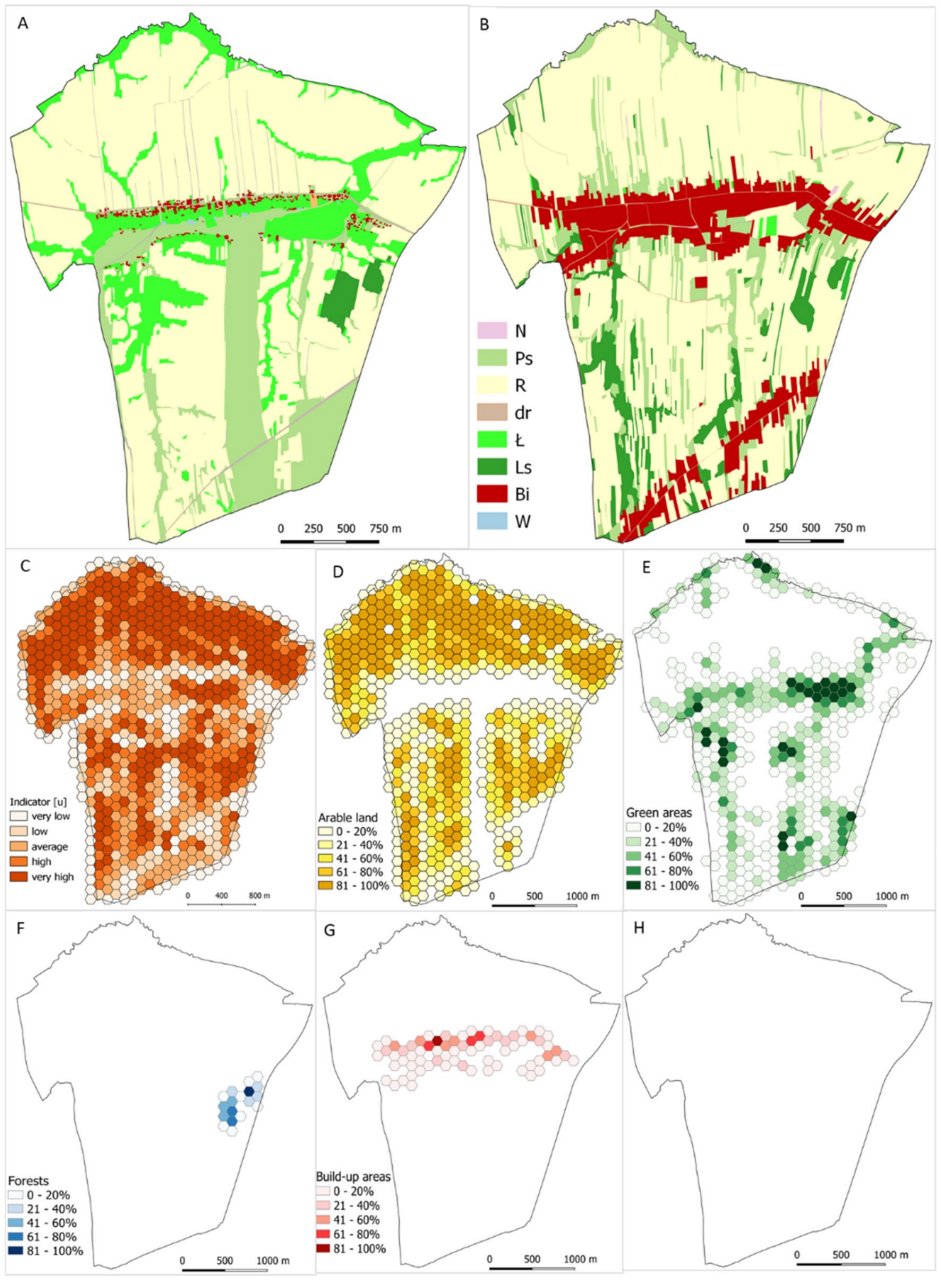
Stages of the spatial assessment of land-use (land-cover types) structure persistence analysis. (**A**) Land use structure in the start time ***t***_**0**_; (**B**) Land use structure in the end time ***t***_**1**_; (**C**) Land-use structure persistence index ***u*** spatial distribution; (**D**) persistence of arable land; (**E**) persistence of grassland; (**F**) persistence of forests; (**G**) persistence of built-up areas; (**H**) persistence of wasteland.

After an assessment of the general trend in land-cover change from 1848 to 2016, it is evident that the area has remained agricultural in nature. Areas of land-use types in land cover changed only in some parts of the village. In 1848, the area of agricultural land was 95.6%, while today it is 76.8% (arable land and grassland). The difference in agricultural land area between the mid-nineteenth century and today (2016) is 18.8%. The dominant land-use type is arable land, which occupies 62.5% of the area, while in the nineteenth century, it was 61.8%. The second land-use type is grassland (meadows and pastures). The spatial assessment of land-cover type persistence (***u***) in Kaszów demonstrated a high level of preservation of the historical structure. As much as 33.8% of the area (262 hexagons) exhibited a very high level of consistency of historical and current use, while 20% of the area exhibits a high level (Fig. 4, compare with Fig. 7G). Only 13% of the area has a very low consistency of less than 20%. The northern part of the area demonstrated the highest persistence of the land-use structure.

### Spatial assessment of agricultural land structure persistence

**Figure 5:**
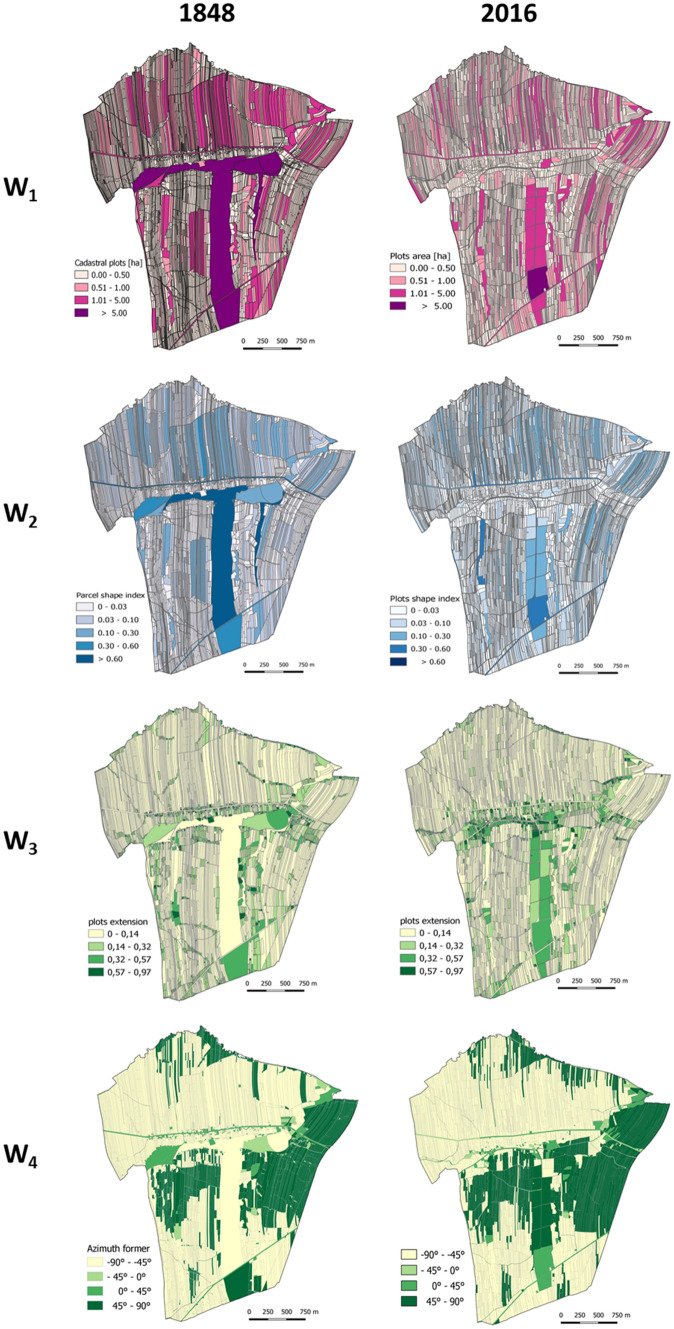
Spatial distribution of component indices.

**Figure 6:**
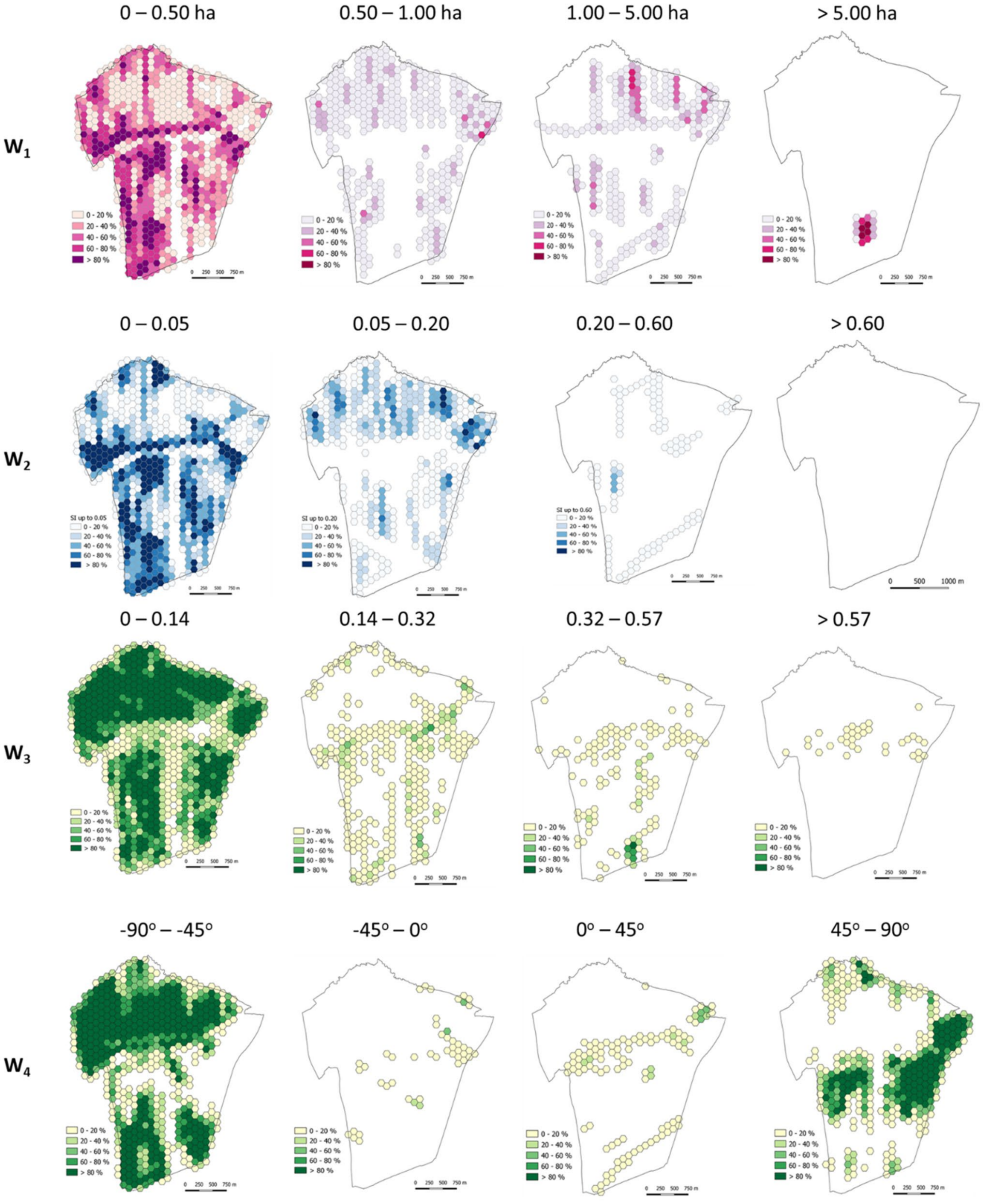
The variation in component indices by variable category.

Spatial variation in component indices of the agricultural land structure (plot area (***w***_**1**_), plot shape (***w***_**2**_), plot elongation (***w***_**3**_), and mean plot axis angle (***w***_**4**_) were obtained. The spatial diversification of the component indices was divided into four classes based on national and regional statistics and literature (Fig. 5, 6).

#### Areas of plots and parcels of land (***w***_1_)

**Figure 7:**
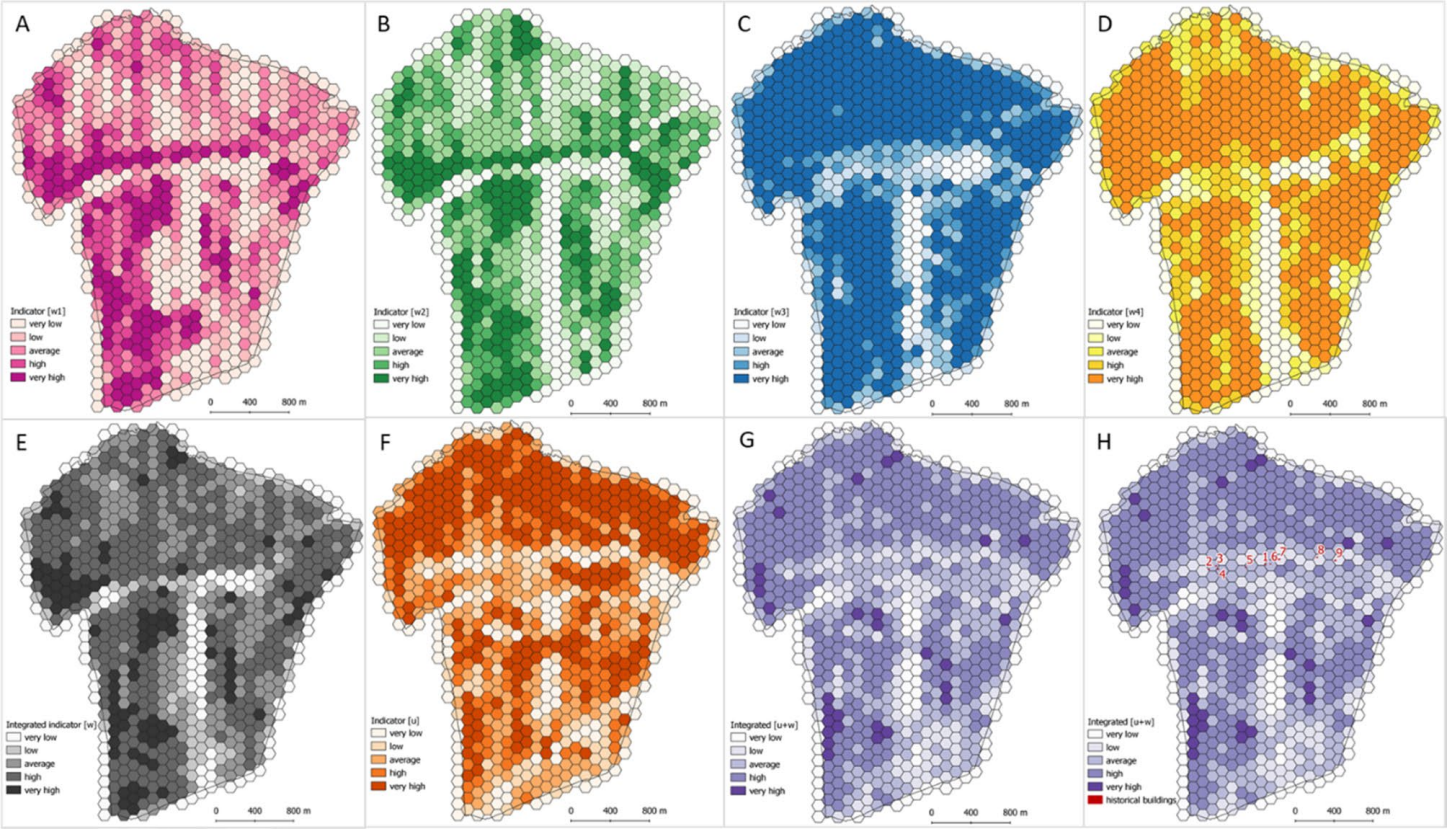
Spatial variation in the persistence of component indices. (**A**) ***w***_**1**_ index; (**B**) ***w***_**2**_; (**C**) ***w***_**3**_; (**D**) ***w***_**4**_, (**E**) land structure persistence integrated index (***w***); (**F**) Land-use structure persistence index ***u;*** (**G**) land structure persistence integrated index (***u*** + ***w***), (**H**) persistence phenomenon integrated index (***u*** + ***w + z***).

Parcels in Kaszów were rather small with the mean value of 0.1742 ha and coefficient of variation 4.9 in 1848. Today, the mean plot area remains small. It is 0.2136 ha with the coefficient of variation of only 1.5. In 1848, Kaszów was subdivided into 3,902 parcels with 91% of them smaller than 0.5 ha. Now, Kaszów has 4,399 plots, and 91% of them are not larger than 0.5 ha (Fig. 7A).

#### Shape index (***w***_2_)

The mean value of the shape index for Kaszów in 2016 r. was 0.019, in 1848, 0.017. In 1848, 91.7% of the parcels exhibited ***w***_**2**_ index up to 0.05, which is similar to today's share of 91.5%. About 8% of the plots had ***w***_**2**_ ranging from 0.05 to 0.20 in time ***t***_**0**_ and ***t***_**1**_ (Fig. 7B).

#### Plot elongation index (***w***_3_)

The plots in Kaszów are usually rather elongated, narrow quadrangles (rectangles, trapeziums). The mean plot elongation in 2016 was 0.154, while in 1848, 0.221. In 1848, 67% of the parcels were elongated up to 0.24 and 20% from 0.24 to 0.48. In 2016, elongation of up to 0.24 was exhibited by 81% of plots, while 12% were elongated from 0.24 to 0.48 (Fig. 7C).

#### Main plot axis angle (***w***_4_)

As many as 62% of plots in Kaszów exhibited a mean plot axis angle located in the –90° to –45° quadrant in 2016. This value was high in 1848 as well, 56%. The smallest number of plots was found in the quadrant ranging from –45° to 0°: 5.5% in 1848 and 2% in 2016 (Fig. 7D).

The variation in the persistence of land structure component indices (***w***_**1**_–***w***_**4**_) in each index was divided into five intervals (20% each).

The most persistent one was index ***w***_**4**_, main plot axis angle. Its spatial persistence is very high for 54.7% of the area. This result demonstrates that directions of plots are the same for both time points for most of the area. Another index with a high level of persistence was plot elongation (***w***_**3**_). Very high and high persistence was found for 58.1% of the area. The area still has plots with similar characteristics, including elongation. The two other features, area and shape, did not exhibit high persistence. Only 16.1% of the area had very high field area persistence. It could suggest a significant number of plot subdivisions over the period. In 1848, the village had 3,902 parcels, while today the number is 4,399. Subdivisions of plots changed their shapes, which is apparent from the shape index, ***w***_**2**_. The area exhibits a rather low spatial persistence of the index. In accordance with the methodology, the integrated index is the mean of the component indices (Fig. 7E). The spatial assessment of the persistence of agricultural land structure (***w***) demonstrated that only 12% of the area exhibited land structure that was very similar to the same forms in the past. The largest share, 43% of the area exhibited high persistence. Very low and low persistence of the land structure was found for 12% and 8% of the area, respectively. The medium persistence was found for 25% of the area. The spatial distribution of the land structure persistence is shown in Fig. 7F. The results show that the land structure of the area changed to a larger or lesser degree. Some plots were subdivided, which increased their number and the mosaic character of the area. Nevertheless, the subdivisions had a moderate impact on the spatial arrangement of the plots because they most often involved dividing the plot perpendicularly to the longest boundary.

**Figure 8:**
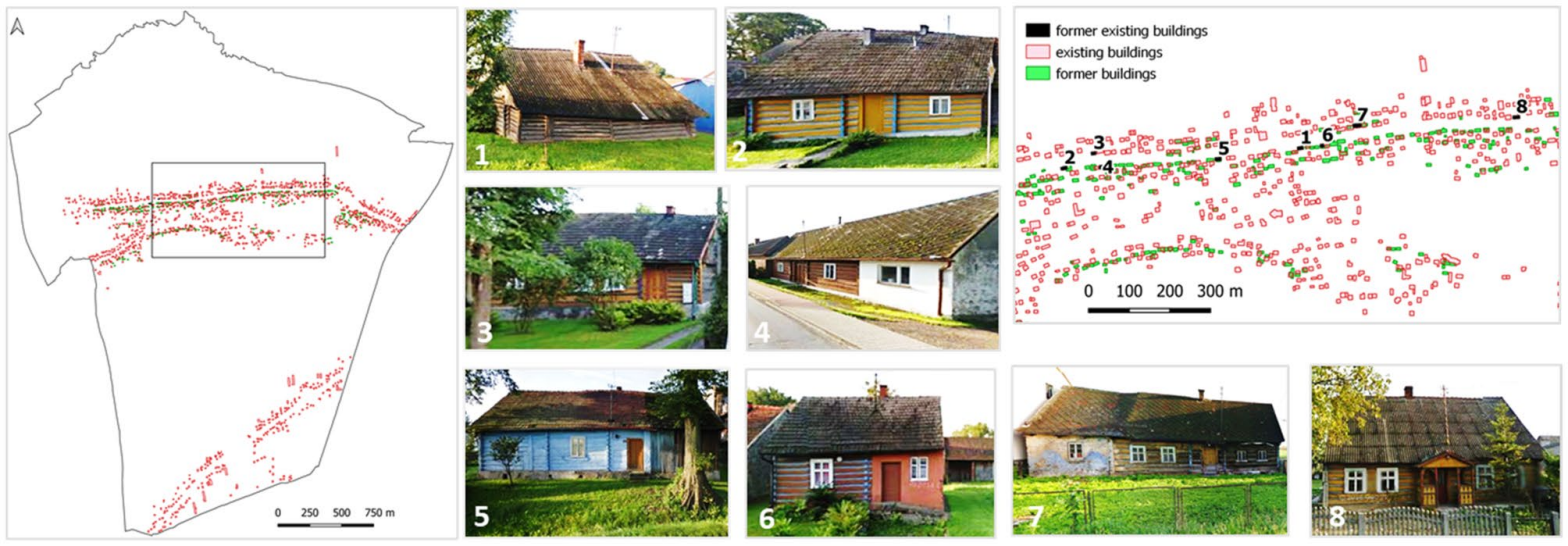
Analysis of historical and modern development overlap; identification of historical objects with examples of historical buildings in Kaszów identified using land structure persistence method.

Figure 8 shows maps with spatial variation in buildings from 1848 to 2016. With Eq. (6), the authors identified buildings that have overlapping parts for the two time points. They pinpointed potential objects that could be dated back to 1848. The analysis of the results and a site visit yielded eight objects in the village that could potentially be dated back to 1848.

The aggregation of indices ***u***, ***w***, and ***z*** yielded the spatial distribution of the persistence of layouts in Kaszów (Fig. 7H). The spatial assessment of layout persistence demonstrated that only 4% of the area exhibited layouts very similar in the two time points. The largest share, 42% of the area exhibited high persistence. Very low and low persistence of the layout structure was found for 11% and 15% of the area, respectively. The medium persistence was found for 28% of the area.

## Discussion

The empirical spatial arrangements evaluation method aims at identifying the persistence of landscape understood as traditional, in the context of rural cultural heritage.

The assessment requires an assumption that the analysed space has striped or patchwork layouts of plots with fields, baulks, ponds, meadows and forests in the landscape structure, which in geographic terms limits the method's applicability to agricultural areas in temperate Central and Eastern Europe.

Traditional landscapes preserved their character thanks to the low dynamics of changes in the past^70^. In general, the literature describes traditional agricultural landscapes as those where farming consumes low external resources and is dominated by relatively small (family) holdings^21^. In structural terms, these landscapes usually have smaller fields and components that reflect past ways of management, such as hedgerows or stone walls^41^. When classifying such landscapes, one should assess the persistence of land-cover types^40^ and persistence of land structure or field layout^23,41^. The degree of persistence of spatial arrangements helps determine the extent to which the landscapes hold cultural heritage and the level of impact of consecutive transformations. The proposed method can help identify traditional agricultural landscapes^2, 37,39^ where the natural environment and farm structure demonstrate an evident mosaic layout. It narrows down the area for analysis by identifying sites with a significant potential for historical spatial arrangements and buildings that could be part of cultural heritage. An additional effect can be the indication of spatial units that were particularly resilient to changes in the landscape structure. In some cases resilience can originate from economic stagnation, as it was in case of Poland during the communist period. On the other hand, historical agricultural reforms in many Eastern Bloc countries irretrievably destroyed the spatial structure of land^10^. As economic growth often causes changes, periods of stagnation and stability tend to promote small-scale adaptation^70^. Sites resistant to transformation should be the focal point of institutions responsible for regional assistance and development.

The study has shown that the structure of the investigated area consists of small fields. It was determined, however, that further modifications have burdened the current sizes of plots and fields as well as their layout. Only part of the area was found to have a high degree of persistence of historical layouts from 1848 (see Fig. 7G). Only four per cent of the study area had the best, very high level of persistence of historical layouts, while 42% exhibited high persistence. This supports the conclusion that one should be particularly cautious when defining traditional historical landscapes in an area, which is consistent with findings by Renes^1^.

The assessment of rural development arrangement persistence was based on the building boundary persistence^55^. It identified potential historical rural developments in the area. There were eight buildings identified that could date back to 1848 (see Fig. 7H).

The proposed methodology helps identify particular historical locations, enclaves of field systems that retained their unique historical spatial structure of the land, with the associated settlement structures in the agricultural landscape of Central and Eastern Europe. Moreover, the method is relatively simple to apply and very cost-effective (economically justifiable).

The proposed method can monitor the current persistence in rural cultural landscapes in regular time intervals. Plenty of rural cultural landscapes face severe threats due to the intensification or abandonment^21^. Knowledge gained through the identification of homogeneous sets of spatial units and the determination of the persistence of local historical spatial arrangements can be applied to planning documents to facilitate adaptation and protection. Renes^1^ noted that an in-depth understanding of a complex history of landscapes could help transform heritage landscape management from the choice between protection or development to a more sophisticated process of change management. The data can further be used when implementing municipal development policies such as the promotion of tourism, protection, or repairs^71^.

## Conclusions

The proposed methodology for determining the persistence of landscape structural layouts employs a GIS tool, intersect of land-cover type, agricultural land layout, and buildings persistence. Land arrangements were assessed with such parameters as the plot area, shape, or elongation and the main plot axis angle directly representing the spatial arrangement of the field layout (referred to in Polish literature as the patchwork). They aggregated the analysed components to identify spatial units exhibiting various degrees of persistence of rural historical spatial arrangements (low to very high). This quantitative approach to the landscape layout structure persistence has not been employed to date with the proposed methodological conditions.

Rural areas are currently under pressure from changes resulting from the introduction of extensive agricultural policies, changes in land use dictated by economic considerations and climate change. Structural transformations taking place in the rural space cause irreversible changes. The article attempts to define the components of cultural heritage related to the spatial structure of land in a comprehensive way. They were quantified and a method of empirical evaluation of landscape durability was proposed. This method allows us to identify places more resistant to transformation and those with greater cultural potential.

The proposed method can identify agricultural areas in Eastern and Central Europe isolated in terms of development that are characteristically located and have a specific local community with features characteristic to it. It is the place Solymosi^2^ indicated when investigating the impact of various factors on the preservation of cultural heritage.

The proposed tool can be used to monitor the degree of changes in the landscape layout structure when multiple time points are analysed as well. The method can also be applied to a shorter period for which maps with use or ownership structure are available, such as from the early twentieth century or after the Second World War.
